# Improvements in the utilization of calcium carbonate in promoting sustainability and environmental health

**DOI:** 10.3389/fchem.2024.1472284

**Published:** 2024-10-03

**Authors:** Jackson Comes, Emir Islamovic, Carlos Lizandara-Pueyo, Jong Seto

**Affiliations:** ^1^ School for the Engineering of Matter, Transport, and Energy, Center for Biological Physics, Arizona State University, Tempe, AZ, United States; ^2^ BASF Corporation, Raleigh, NC, United States; ^3^ BASF SE, Ludwigshafen, Germany; ^4^ Molecular Foundry, Lawrence Berkeley National Laboratory, Berkeley, CA, United States

**Keywords:** calcium carbonate, catalysis, carbon capture, agriculture, inorganic materials chemistry

## Abstract

Calcium carbonate (CaCO_3_) is an incredibly abundant mineral on Earth, with over 90% of it being found in the lithosphere. To address the CO_2_ crisis and combat ocean acidification, it is essential to produce more CaCO_3_ using various synthetic methods. Additionally, this approach can serve as a substitute for energy-intensive processes like cement production. By doing so, we have the potential to not only reverse the damage caused by climate change but also protect biological ecosystems and the overall environment. The key lies in maximizing the utilization of CaCO_3_ in various human activities, paving the way for a more sustainable future for our planet.

## Introduction

**FIGURE 1:**
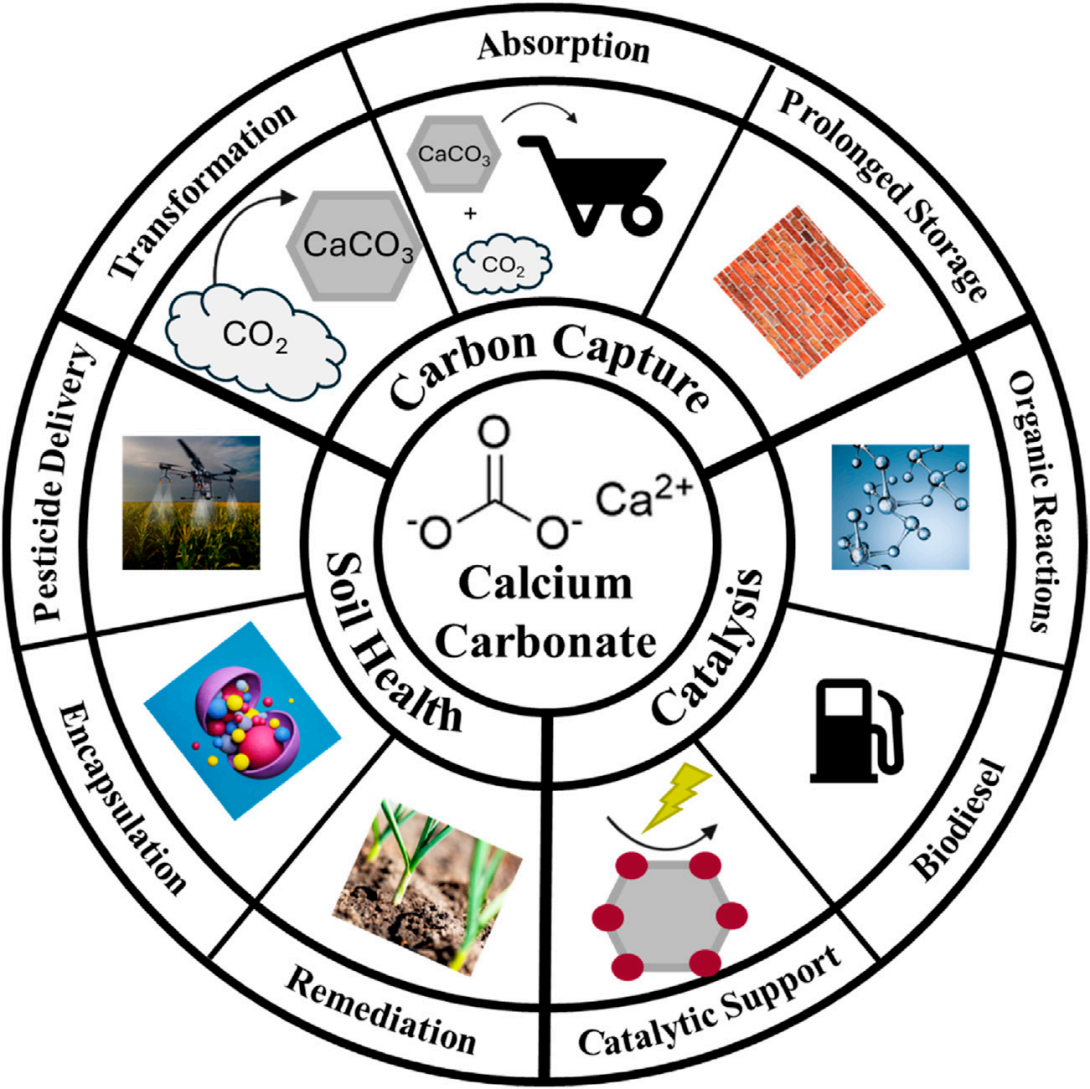
Schematic representation of current uses of calcium carbonate discussed in this mini review.

The significance of inorganic materials in the chemical industry cannot be overstated. These materials, derived from non-carbon-based compounds, play a foundational role in various processes, applications, and innovations (Gągol et al., 2020; Industrial inorganic chemistry, 2010; Wang and Wang, 2021; Wang et al., 2022; Waris et al., 2021) (Figure 1). As we navigate an era emphasizing sustainability, the importance of inorganic materials in fostering sustainable chemistry becomes increasingly apparent (Huang and Zhai, 2021; Lima et al., 2020; Van Soest et al., 2021). Inorganic materials serve as essential building blocks for countless chemical products, ranging from catalysts and reagents to structural components (Clark and Rhodes, 2000; Furukawa and Komatsu, 2017; Mitzi, 2009; Osterloh, 2008; Schubert and Hüsing, 2019; Song and Lee, 2002; Zheng et al., 2022). Their versatility extends into diverse sectors such as electronics, pharmaceuticals, energy, technology and materials science (Al Zoubi and Ko, 2020; Avouris and Martel, 2010; Boles et al., 2016; Chen and Park, 2018; Chen et al., 2015; Ebadi Jamkhaneh et al., 2021; Fadia et al., 2021; Fan et al., 2021; Moon et al., 2007; Niemeyer, 2001; Qi et al., 2020; Servin and White, 2016; Sun and Rogers, 2007; Vallet-Regí et al., 2007).

Crucially, in the context of sustainable chemistry, inorganic materials contribute to environmentally conscious practices (Caballero-Calero et al., 2021; Mazari et al., 2021; Nelson and Schelter, 2019; Pham et al., 2020). Their role in catalysts (Boles et al., 2016; K.-G; Liu et al., 2021; Shen et al., 2020) and processes designed for cleaner and more energy-efficient production and applications (Kitchen et al., 2014; Zheng et al., 2022) exemplifies their significance. Additionally, their use in energy storage (Cheng et al., 2021; Junaid et al., 2021; Liu et al., 2020; Piątek et al., 2021), renewable energy technologies (Chandrasekaran et al., 2011; Liang et al., 2017), and waste treatment (Goh and Ismail, 2018; Kayvani Fard et al., 2018; Manikandan et al., 2022; Xiang et al., 2022) underscores their pivotal role in addressing global sustainability challenges.

Calcium carbonate serves as a versatile reagent in inorganic chemistry, contributing to various reactions and processes (Al-Hosney and Grassian, 2004; Baltrusaitis et al., 2007; Lin et al., 2020; Salek et al., 2015; Suppes et al., 2001). Moreover, its unique properties such as its porous structure and high surface area (Durand et al., 2018), make it a suitable substrate for anchoring catalytically active components (García-Mota et al., 2011; Liu et al., 2013; Saetan et al., 2017; Schlägl et al., 1987). Calcium carbonate plays a role in the production of biodiesel, catalyzing transesterification of natural oils (Alonso et al., 2010; Chutia and Phukan, 2024; Kouzu et al., 2008; Ling et al., 2019; X; Liu et al., 2021; Ngamcharussrivichai et al., 2010; Suppes et al., 2001; Thangaraj et al., 2019). Calcium carbonate is employed in carbon capture applications as a sorbent for CO_2_ removal (Dou et al., 2016; Erans et al., 2016; Florin et al., 2010; Liu et al., 2010; Witoon, 2011). In a process called mineral carbonation, it reacts with carbon dioxide to form stable carbonates, contributing to carbon capture and storage efforts (Abanades, 2002; Bewernitz et al., 2024; Erans et al., 2020; Gadikota, 2021; Gambhir and Tavoni, 2019; Levey et al., 2024; Olajire, 2013; Sanna et al., 2014; Sanz-Pérez et al., 2016). Like in biological materials, various mineral phases of CaCO_3_ can be processed to obtain customized chemical reactivity and functionality (Briegel and Seto, 2012; Cho et al., 2019; Seto et al., 2014; 2013). This method aids in mitigating greenhouse gas emissions and addresses climate change concerns (Neeraj and Yadav, 2020; Snæbjörnsdóttir et al., 2020; Thonemann et al., 2022). Understanding these diverse properties unveils calcium carbonate’s significance in addressing environmental concerns.

This material emerges as a linchpin for fostering robust soil health, vibrant plant growth, and bountiful crop yields (Hamdan et al., 2017; Soon et al., 2014; Wang et al., 2015; Xie et al., 2024). From soil pH adjustment in acidic terrains (Neina, 2019) to serving as a vital calcium supplement for plants (Shabtai et al., 2023), calcium carbonate’s agricultural significance is underscored by its ability to rectify deficiencies that may impede the optimal development of crops. Furthermore, its impact extends beneath the surface, where it actively participates in enhancing soil structure (Dou et al., 2023). By promoting aggregation, calcium carbonate facilitates improved water retention and drainage, creating an environment conducive to the flourishing of roots (Figure 1).

As an additive in fertilizers, it takes on the role of a nourishing component, supplying essential calcium that supports the formation of robust cell walls and overall plant structure (Abo-Sedera, 2016; Hua et al., 2015). Acting as a buffering agent, calcium carbonate becomes a guardian of soil pH stability, curbing rapid fluctuations that could detrimentally affect plant health (McFarland et al., 2020; Zhang et al., 2016). Beyond the crop fields, its practical application even extends to dust control in agricultural settings, where it contributes to creating a more comfortable environment, particularly in livestock farming (Hamdan and Kavazanjian, 2016; Meyer et al., 2011; Song et al., 2020). In essence, the diverse applications of calcium carbonate in agriculture stand as a testament to its integral role in promoting soil fertility, sustaining healthy plant growth, and ultimately cultivating agricultural landscapes that thrive (Figure 1).

## CaCO_3_ in carbon capture and mineralization applications

**FIGURE 2:**
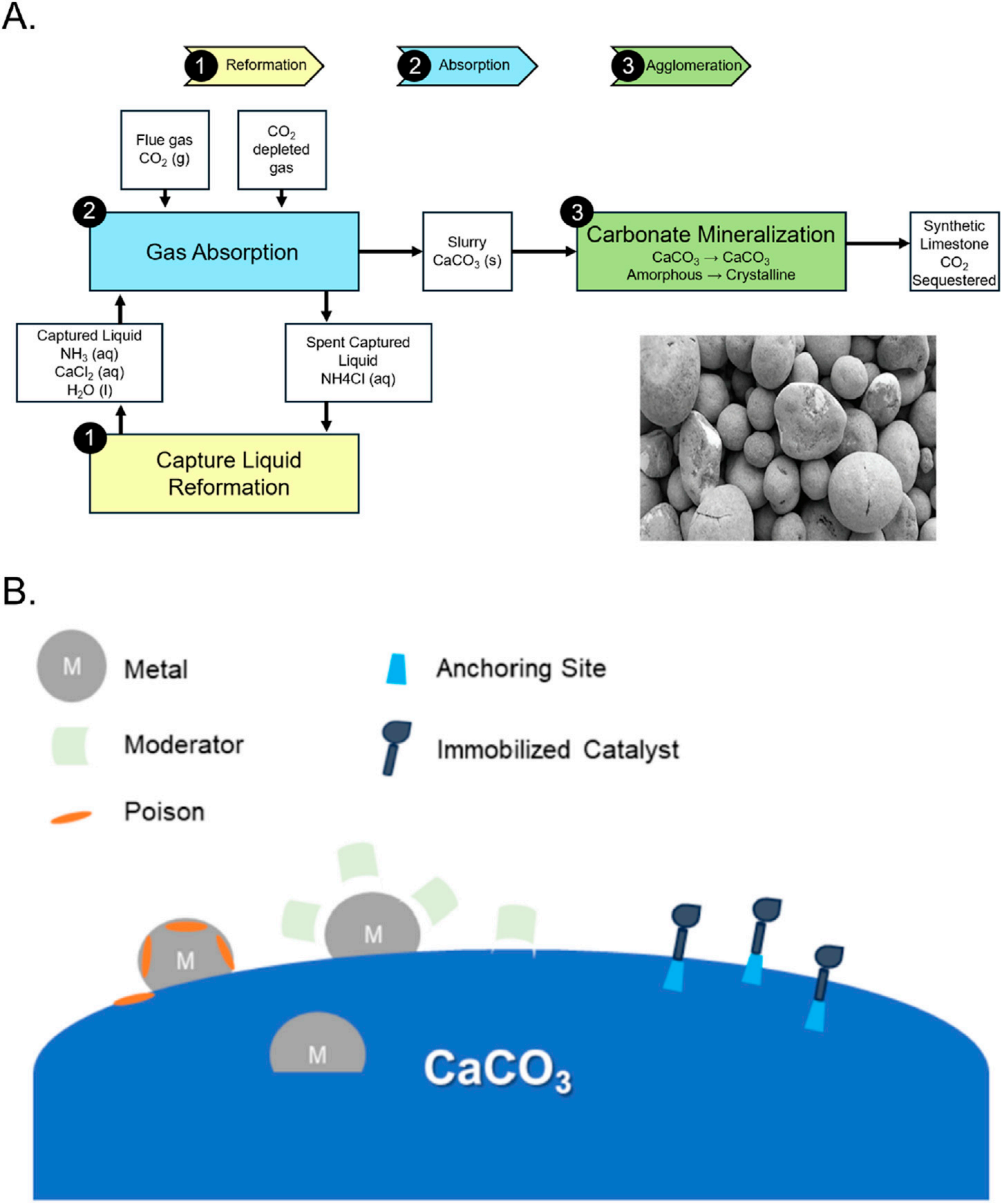
Diverse methods of utilizing CaCO_3_ at various length-scales. **(A)** By utilizing a biogeomimetic mineralization route, recycled geomass can be harvested to make tonnes of aggregates. [inset: CaCO_3_ aggregates formed] (images courtesy of Blue Planet Systems, Inc). **(B)** CaCO_3_ can serve as catalysts with specific functional groups to enhance the formation of chemical feedstocks. (conceptual graph inspired by, among others, Lindlar and Dubuis, 2003; Senra et al., 2008; Lizandara-Pueyo et al., 2021).

Carbon capture, utilization, and storage (CCUS) technologies aim to decrease the greenhouse gas effect by capturing emitted carbon and transforming it for long term storage or chemical utility (Chang et al., 2017). Industrial mineralization of carbon dioxide to produce calcium carbonate is a promising CCUS method with high economic potential (Chang et al., 2017; Teir et al., 2016). These reaction pathways valorize waste streams from processes such as steelmaking and cement production while reducing energy consumption (Jin et al., 2022; Katsuyama et al., 2005; Marin Rivera and Van Gerven, 2020; Teir et al., 2016). By utilizing chemicals in waste flue gas, steelmaking slag and cement powder, calcium carbonate production provides a green alternative to disposal and storage of carbon dioxide (Czaplicka and Konopacka-Łyskawa, 2020; Teir et al., 2016) (Figure 2). There are many methods to precipitate calcium carbonate from CO_2_ streams such as microbially induced precipitation, ultrasonication of supercritical carbon dioxide, and other methods, most of which are based on biomimetics or CO_2_ bubbling (Boyjoo et al., 2014; Chuo et al., 2020; López-Periago et al., 2010). When using sorbent technology, a compromise must be made between sorbent performance and the increasing cost (Erans et al., 2016). Modified materials and advanced chemical reactors increase sorbent utility, but can be vastly more expensive than their simpler counterparts. Also, the activity of sorbents decay over time due to sintering and attrition, further limiting utility (Erans et al., 2016).

In recent years, chemical looping and mineralization has been gaining attention as a promising CCSU technology, with publications such as those from Jin, Z. et al. and Bewernitz, M.A. et al. (Table 1). CaCO_3_ aggregates can be used to replace cementitious products (Hargis et al., 2021; Pu et al., 2021). Through an exponential increase in the built environment, cement production has become an ever-increasing source of CO_2_ and dust pollution. It approximately accounts for 8% of the anthropogenic CO_2_ produced per year and is a process with very little technical improvement since its utilization from Roman times (Stefaniuk et al., 2023). Only through recent advances like replacements with clinker as well as new formulations with lower energy substitutes (Martinez et al., 2023) as well as aggregates (Hanifa et al., 2023). can we envision a world with more infrastructure, but without the pollution attached to building it (Figure 2).

**TABLE 1:** Publications related to calcium carbonate sustainability arranged by year.

Application	Topic	Scope	Year published	Author(s)
Carbon capture	Transformation	Carbonation	2002	Abanades, J.C
		Sorbent	2010	FlorinN.H.
		Sorbent	2010	Liu, W. et al
		Sorbent	2019	Gambhir, A
		MICP	2020	Chuo, S.C. et al
		Carbonation	2020	Czaplicka, N. et al
		Carbonation	2020	Yadav, S
		Mineralization	2020	Marin Rivera, R. et al
		Mineralization	2021	Gadikota, G
		Chemical looping	2022	Jin, Z. et al
		Liquid condensed phase	2024	Bewernitz, M.A. et al
	Absorption	Biomineralization	2013	Dhami, N.K et al
		Sorbent	2016	Erans, M
		Carbonation	2016	Sanz-Pérez, E.S. et al
		Sorbent	2020	Erans, M
	Prolonged storage	Scale up	2011	HerzogH.J.
		Carbonation	2017	Chang, R. et al
		Carbonation	2020	Snæbjörnsdóttir, S.Ó. et al
		Carbonation	2021	Campo, F.P et al
		Cement	2021	Hargis, C.W. et al
		Cement	2023	Hanifa, M. et al
		Cement	2024	Levey, C. et al
Catalysis	Biodiesel	Transesterification	2008	Kouzu, M. et al
		Transesterification	2010	Alonso, D.M. et al
		Transesterification	2010	Ngamcharussrivichai, C. et al
		Transesterification	2010	Liu, X. et al
		Transesterification	2024	Chutia, G.P. et al
	Catalytic support	Selective hydrogenation	1987	Schlägl, R. et al
		Selective hydrogenation	2008	Senra, J.D. et al
		Selective hydrogenation	2011	García-Mota, M. et al
		Cross coupling reactions	2013	Liu, H. et al
		Cross coupling reactions	2017	Saetan, T. et al
		Selective hydrogenation	2020	Laverdura, U.P. et al
		Asymmetric Michael addition	2021	Lizandara-Pueyo, C. et al
		Selective hydrogenation	2022	Ballesteros-Soberanas, J. et al
	Organic reactions	Alcoholysis	2001	Suppes, G.J. et al
		Intermediate surface reactions	2004	Al-Hosney, H.A. et al
		Sulfur dioxide reactions	2007	Baltrusaitis, J. et al
Soil health	Pesticide delivery	Nanoparticles	2018	Zhao, X. et al
		Fungicide	2022	Zhou, Z. et al
		Sporopollenin	2023	Xiang, S. et al
	Encapsulation	Sunflower pollen	2016	Mundargi, R.C. et al
		Slow release	2023	Abhiram, G. et al
	Remediation	Pond soil	2004	Queiroz, J.F.D. et al
		MICP	2011	Meyer, F.D. et al
		MICP	2014	Soon, N.W. et al
		MICP	2020	Song, J.Y. et al
		pH control	2015	Salek, S. et al
		pH control	2016	Juang, Y. et al
		pH control	2018	Gentili, R. et al
		pH control	2020	McFarland, C. et al
		Foliar spray	2016	Abo-Sedera, F
		Dust control	2016	Hamdan, N. et al
		Denitrification	2017	Hamdan, N. et al
		Improved tomato yield	2018	Patanè, C. et al
		Metal remediation	2019	Bashir, M.A. et al
		Metal remediation	2020	Lin, P.-Y. et al
		Cauliflower development	2020	Santos, C.A.D. et al
		Carbon regulation	2023	Dou, X. et al
		Improved wheat yield	2023	Gao, Y. et al
		Pathogen elimination	2023	Liu, Q. et al

*MICP, microbially induced calcium carbonate precipitation.

**FIGURE 3:**
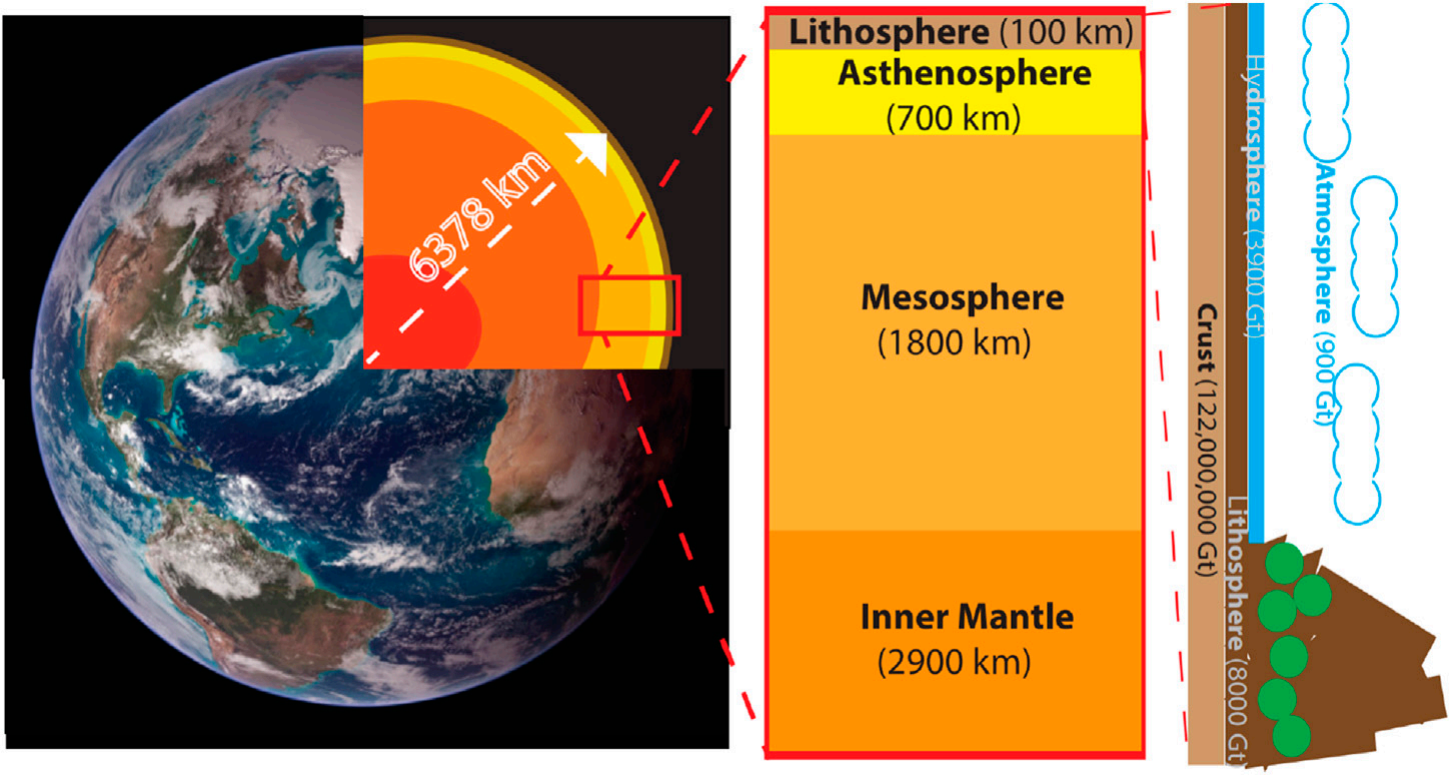
The geological composition of the Earth **(A)**. The various segments from the core to the surface of the planet **(B)**. The diverse composition of the Earth’s mantle **(C)**. The majority of carbon (in the form of carbonates and CO_2_) stored in the diverse surface layers of Earth (image courtesy of NASA).

It is indisputable that the indiscriminate emissions of greenhouse gas have resulted in increased surface temperature on Earth and environmental degradation (Yoro and Daramola, 2020). Given the tremendous amount of CO_2_ in Earth’s atmosphere, CCUS technology would have to make gigaton-scale changes to have a meaningful impact on the global scale (Figure 3). Through reduction of emissions and increasing global CCUS usage, humanity has been trying to reduce the impact of climate change caused by anthropogenic CO_2_ emissions (Dey and Dhal, 2019; Huisingh et al., 2015; Sanna et al., 2014).

The relationship between average surface temperature and CO_2_ concentration is directly proportional (Humlum et al., 2013). Ice core records indicate that CO_2_ concentration has varied with temperature over long time scales for the past 420,000 years or even longer (Humlum et al., 2013; Lüthi et al., 2008). Also, it is suggested that rising atmospheric CO_2_ levels amplify or even precede global temperature changes initiated by Milankovitch cycles (Humlum et al., 2013; Shakun et al., 2012; Toggweiler and Lea, 2010). With these facts in mind, data from the National Center for Environmental Information (NOAA National Centers for Environmental Information, 2024; U.S. Global Change Research Program et al., 2017) was plotted and regression was performed to derive Equation 1, describing the relationship between global average surface temperature (as compared to the 1901–200 average), time, and atmospheric CO_2_ concentration.

$$T=\left(1-\alpha \times t\right)\left(3.5\times 10^{-26}\right)\exp\left(0.029\times t\right)$$ (1)

**FIGURE 4:**
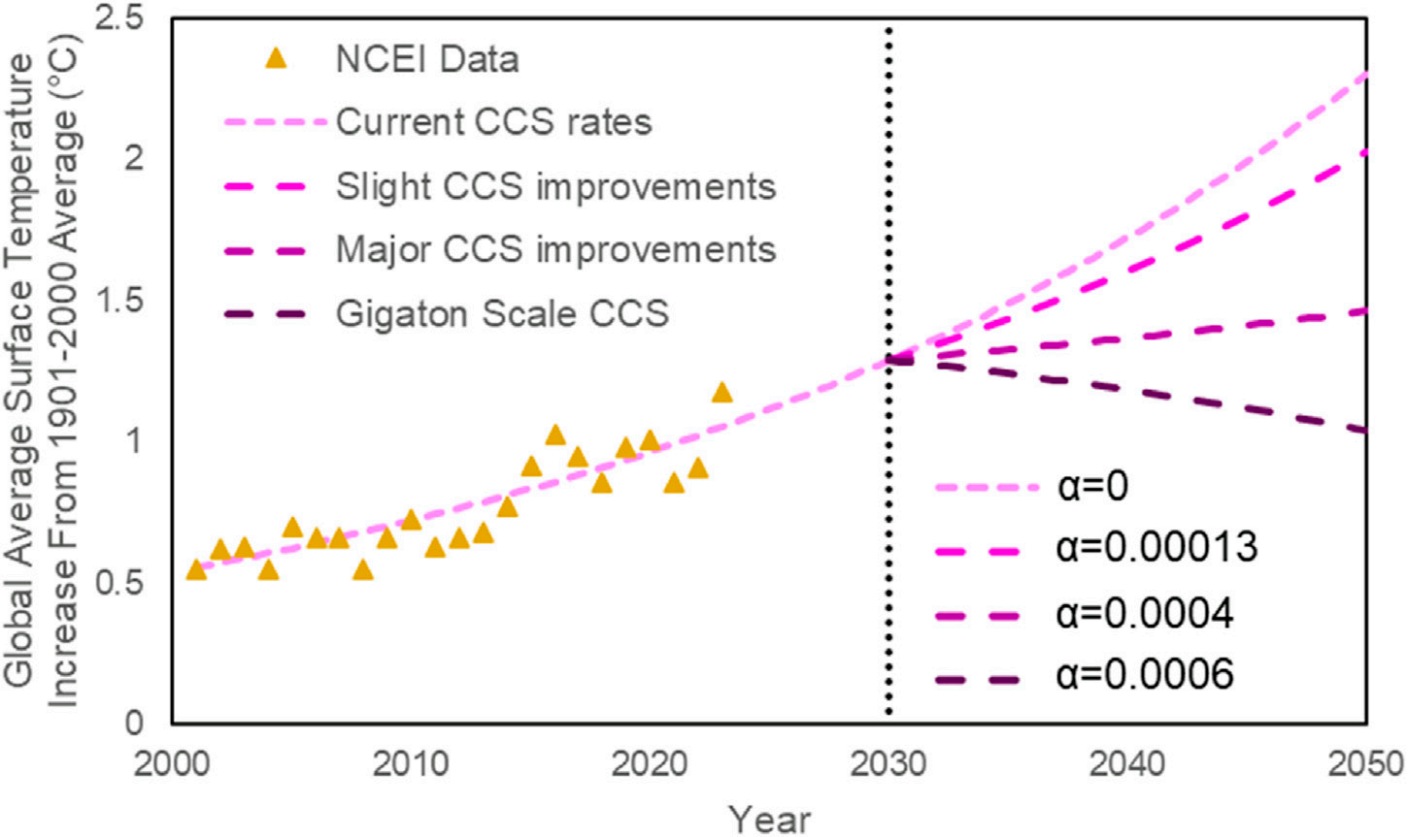
Global average surface temperature increase from 1901 to 2000 average based on data from the National Centers for Environmental Information (triangles) (NOAA National Centers for Environmental information, 2024).

The factor $\left(1-\alpha \times t\right)$ in Equation 1 accounts for humanity’s intervention and efforts to halt the rising global temperatures. The variable ⍺ is proportional to the amount of CO_2_ being captured and removed from the atmosphere. With a higher amount of CCS technology used globally, α would increase proportionally. If no changes are made to humanity’s current emission rates, $\left(1-\alpha \times t\right)=1$ , and the current trend is expected to continue, increasing surface temperatures and further harming global ecosystems (Ainsworth et al., 2020; Karnosky, 2003; Moore et al., 2021; Prakash, 2021; U.S. Global Change Research Program et al., 2017). Improving CCS technology has the potential to slow this temperature increase, but not enough to see impactful differences at current rates (Davis, 2017). Even if carbon dioxide emission was completely halted, and all emitted CO_2_ were captured and stored, the Earth’s temperature is expected to remain the same or continue increasing at a very slow rate for centuries (Frölicher et al., 2014). Such a carbon neutral state can only be accomplished through radical improvements to CCS technologies, and drastically decreasing reliance on fossil fuels (Budinis et al., 2018; Stone et al., 2009). CCS technology will only become a reasonable solution to rising surface temperatures through sequestering billions of metric tons (gigatons) of carbon dioxide per year (Herzog, 2011; Valone, 2023) (Figure 4).

Given Earth’s tremendously large surface area, and high water content, it takes an enormous amount of energy to raise the average surface temperature even by a small amount. Though 1°C may seem like an insignificant change, the global effects are drastic due to the tremendous amount of trapped heat (Lindsey and Dahlman, 2024; NOAA National Centers for Environmental Information, 2024; U.S. Global Change Research Program et al., 2017). If dramatic industrial and legal changes are not made, the disastrous effects may be irreversible (Armstrong McKay et al., 2022; Ridley et al., 2010).

## CaCO_3_ in chemical catalysis

One of the most relevant processes in the chemical industry is the selective partial hydrogenation of alkynes to alkenes; this transformation is usually enabled by the Lindlar Catalyst (Lindlar and Dubuis, 2003). The use of calcium carbonate in the Lindlar Catalyst dates back to the 1980s when authors such as Schlägl, R. et al. released publications detailing its structure and activity (Table 1). This catalyst is based on palladium supported on calcium carbonate and passivated with lead acetate and quinoline. This catalytic system has a broad application in natural product synthesis (Ballesteros-Soberanas et al., 2022) or in the upgrading of vegetable oils (Laverdura et al., 2020). This catalyst allows the reaction to be performed at high temperatures (200°C), increasing conversion rates while simultaneously increasing the cost required to run such a system (Suppes et al., 2001). The versatility and inertness of calcium carbonate as support, allows the fine tuning of the surfaces. Accordingly, an efficient and selective catalytic system using hydroxypropylated cyclodextrins and palladium on calcium carbonate has been presented by Senra et al. (2008) for a ligand-free aqueous Heck reactions. In this case, the catalyst was not only active and selective but also immobilized. Most recently, calcium carbonate has been used as a support for the immobilization of asymmetric catalysts using biomineralization concepts and click chemistry. A calcium carbonate-supported α,α-diarylprolinol silyl ether prepared in this manner catalyzed Michael addition of aldehydes to trans-β-nitrostyrenes with very high diastereo- and enantioselectivity. By utilizing calcium carbonate as a heterogenous support, organocatalysis can be immobilized which reduces the cost and energy requirement of downstream separations (Lizandara-Pueyo et al., 2021). Additionally, this method allows for the used organocatalysis to be recycled and reused (Benaglia, 2009) (Figure 2). The catalyst can be recovered for reuse by simple decantation or used in a continuous flow reactor, increasing productivity five-fold as compared to batch processes (Lizandara-Pueyo et al., 2021).

Recycling catalysts is a sustainable process, but sometimes a certain portion of the catalyst cannot be recovered. A small amount of the catalyst can dissolve into the reaction mixture, and potentially escape into the environment. Calcium is extremely common in the environment as compared to other catalytic chemicals. The miniscule amounts of calcium carbonate which escape into the environment would generally cause no effect because calcium is already present (Suppes et al., 2001).

Remarkably, calcium carbonate is highly recyclable and utilization of recycled calcium carbonate enables a boost in the recycled content found in compounds and final polymer applications, thereby aiding customers in achieving their circular economy objectives. Utilization of pre-consumer and post-consumer recycled calcium carbonate is on rise thus adding another benefit of calcium carbonate as a part of a product (Cunningham et al., 2021).

## CaCO_3_ in agricultural applications

With an increasing global population (Gu et al., 2021), food security is of the utmost importance (Molotoks et al., 2021). Between 2010 and 2050, there is an anticipated increase in global food demand ranging from 35% to 56%. Simultaneously, the population at risk of hunger is projected to undergo a change ranging from a reduction of 91% to an increase of 8% during the same period (Van Dijk et al., 2021). The looming threat of food scarcity heightens humanity’s need to protect and ensure a bountiful supply of healthy crops in the coming years (Anderson et al., 2020; Devaux et al., 2021; Fukase and Martin, 2020; Hasegawa et al., 2021; Zhao et al., 2021). Calcium carbonate offers various solutions to some issues farmers are facing (Liu et al., 2023; Patanè et al., 2018; Santos et al., 2020). By utilizing the unique chemical, physical and material properties of CaCO_3_, humanity can nourish the agricultural industry in a sustainable way, while potentially avoiding the projected food insecurities.

Soils play a vital role in meeting the food and feed requirements of an expanding global population. The addition of calcium carbonate to soil can have a beneficial effect on plant health. Traditionally, it is commonly used as a liming agent to reduce soil acidity, meaning that calcium carbonate acts as a neutralizing agent for acidic soils by increasing the soil’s pH level, making it more suitable for plant growth. Furthermore, it helps to enhance soil aggregation, water retention, and nutrient availability, making the soil more conducive to plant growth. Interestingly, doping soil with calcium carbonate has alleviated poisoning caused by heavy metal pollution in Hunan, China (Zeng et al., 2015). A field study conducted in Tehsil Lahore City, Punjab, Pakistan further confirmed that concentrations of heavy metals were negatively correlated with calcium carbonate concentrations (Bashir et al., 2019).

Calcium carbonate is often used as a filler in fertilizers to improve their physical properties. It helps to prevent caking and improve the flowability of granular fertilizers, making them easier to handle and apply (Abhiram et al., 2023). Calcium is an essential nutrient for plant growth and development. Calcium carbonate is used as a source of calcium to supplement the soil and provide plants with this vital nutrient. It helps in strengthening the cell walls of plants, improving their overall structure and resilience (Gao et al., 2023).

Plant diseases have a substantial effect on crop yields and quality, leading to considerable economic losses and requiring significant management inputs each year for crops, landscapes, and forests in the United States, amounting to billions of dollars (Fones et al., 2020; Ristaino et al., 2021). Plant sporopollenin has recently emerged as an environmentally friendly drug carrier (Mundargi et al., 2016). The sporopollenin capsule modified with calcium carbonate can be loaded with drugs or pesticides and used for controlled release of pesticide (Xiang et al., 2023). The engineered sporopollenin can then be mixed with soil used to grow plants. This process allows for the slow release of drugs, pesticides and calcium carbonate while remaining environmentally friendly and increasing crop yield.

Microcapsule based controlled release formulations are promising alternatives to conventional pesticide. By preparing double shelled calcium carbonate capsules, many adverse effects caused by traditional pesticide can be avoided. Approximately 90% of the liquid-based pesticide is lost to run-off and evaporation (Zhao et al., 2018). The controlled release of pesticides through microcapsules is a promising method to solve such issues (Zhou et al., 2022). Though environmentally favorable, the preparation of pesticide loaded calcium carbonate microcapsules is expensive, due to the large amount or organic substances required (Zhou et al., 2022). Therefore, developing a cheap and efficient production process is needed.

Starch doped porous calcium carbonate can also be used as a pesticide delivery mechanism. Calcium carbonate microspheres fabricated through coprecipitation regulated by soluble starch can be used as drug carriers for plants (Xiang et al., 2018). Porous calcium carbonate microspheres with intercalated soluble starch molecules were used as carriers for Prometryn, a typical herbicide (Xiang et al., 2018). Due to electrostatic attractions and hydrogen bonding, the herbicide is slowly released, controlling migration of the chemical while increasing its utility (Xiang et al., 2018). This method can potentially be expanded to include the use of other chemical herbicides as well.

For agricultural applications, the approval and regulations regarding calcium carbonate microcapsules depend on the specific application and the country in which it is being used. In general, calcium carbonate itself is commonly used and considered safe for various agricultural applications. However, if calcium carbonate is used as a carrier or encapsulating agent for other substances, such as pesticides or fertilizers, the regulations may vary. Nevertheless, this application will be more explored and it will gain more interest in the future in comparison to more traditional calcium carbonate applications (Table 1).

Regulatory bodies, such as the Environmental Protection Agency (EPA) in the United States or the European Chemicals Agency (ECHA) in the European Union, assess and approve the use of carriers and encapsulating technologies based on their specific applications and potential risks. These bodies evaluate factors such as the effectiveness, safety, and environmental impact of the carried or encapsulated substances. Calcium carbonate is a naturally occurring mineral that is not derived from plastic and does not pose the same environmental concerns as microplastics. Calcium carbonate is biologically and chemically distinct from plastics and is not classified as a microplastic.

Naturally, calcium carbonate exists in the form of limestone, a rock that contains a minimum of 50% calcium carbonate. Limestone deposits can be found worldwide and are extracted through quarrying or mining processes. The United States stands as a prominent producer of calcium carbonate, with minimal risk of supply disruption. Presently, the cost of lime for agricultural applications, like non-irrigated corn farming in South Georgia, stands at $55 per ton, amounting to $13.73 per acre or $0.16 per bushel. Although this may appear relatively high, investing in lime can yield returns within two to 3 years (University of Georgia, 2024). However, one should be careful in applying lime in the field as the pH of the soil is an important factor that can be regarded as a crucial variable because of its impact on various other soil properties and processes that ultimately affect the growth of plants. The activity of microorganisms, as well as the solubility and availability of nutrients, are among the vital processes that rely on soil pH. For example, pH has a significant influence on various plant characteristics or traits, including height, lateral spread, biomass, flower size and quantity, pollen production, and more (Gentili et al., 2018; Jiang et al., 2017).

## Conclusion

CaCO_3_ is a mineral with a diversity of utilization throughout the built environment. With increasing atmospheric CO_2_ to ocean and soil acidification, methods to mitigate pollution are ever more required for a sustainable environment. Specifically, these activities include CO_2_ sequestration, chemical catalysis and utilization, precise agricultural applications and essential soil amendments, as well as formation of light weight aggregates in cement replacement applications; all of which will provide noticeable improvements for a more sustainable future. We show an evolution of diverse applications that have been and are using CaCO_3_ however, with time these applications with CaCO_3_ will also multiply and its utility will be invaluable in the near future.
